# Analytical Parameters of a Novel Glucose Biosensor Based on Grafted PFM as a Covalent Immobilization Technique

**DOI:** 10.3390/s21124185

**Published:** 2021-06-18

**Authors:** Margalida Artigues, Joan Gilabert-Porres, Robert Texidó, Salvador Borrós, Jordi Abellà, Sergi Colominas

**Affiliations:** 1 Electrochemical Methods Laboratory-Analytical and Applied Chemistry Department at Institut Químic de Sarrià, Universitat Ramon Llull, Via Augusta, 390, 08017 Barcelona, Spain; margalida.artigues@iqs.url.edu (M.A.); jordi.abella@iqs.edu (J.A.); 2 Tractivus SL, Via Augusta, 394, 08017 Barcelona, Spain; joan.gilabert@tractivus.com (J.G.-P.); robert.texido@tractivus.com (R.T.); salvador.borros@iqs.url.edu (S.B.); 3 Grup d’Enginyeria de Materials (GEMAT) at Institut Químic de Sarrià, Universitat Ramon Llull, Via Augusta, 390, 08017 Barcelona, Spain; 4 CIBER-BBN, Networking Center on Bioengineering, Biomaterials and Nanomedicine, 500018 Zaragoza, Spain

**Keywords:** bioanalytical methods, electrochemical biosensor, hydroxyethyl methacrylate (HEMA), pentafluorophenyl methacrylate (PFM), glucose oxidase (GOx)

## Abstract

Bioanalytical methods, in particular electrochemical biosensors, are increasingly used in different industrial sectors due to their simplicity, low cost, and fast response. However, to be able to reliably use this type of device, it is necessary to undertake in-depth evaluation of their fundamental analytical parameters. In this work, analytical parameters of an amperometric biosensor based on covalent immobilization of glucose oxidase (GOx) were evaluated. GOx was immobilized using plasma-grafted pentafluorophenyl methacrylate (pgPFM) as an anchor onto a tailored HEMA-co-EGDA hydrogel that coats a titanium dioxide nanotubes array (TiO_2_NTAs). Finally, chitosan was used to protect the enzyme molecules. The biosensor offered outstanding analytical parameters: repeatability (RSD = 1.7%), reproducibility (RSD = 1.3%), accuracy (deviation = 4.8%), and robustness (RSD = 2.4%). In addition, the Ti/TiO_2_NTAs/ppHEMA-co-EGDA/pgPFM/GOx/Chitosan biosensor showed good long-term stability; after 20 days, it retained 89% of its initial sensitivity. Finally, glucose concentrations of different food samples were measured and compared using an official standard method (HPLC). Deviation was lower than 10% in all measured samples. Therefore, the developed biosensor can be considered to be a reliable analytical tool for quantification measurements.

## 1. Introduction

Biosensors and related bioanalytical tools are increasingly used as complements for standard analytical methods in a large number of applications, such as environmental monitoring [1,2,3], clinical diagnostic [4,5,6], and food analysis [7,8,9]. These devices offer fast and cost-effective measurements that can be made in situ, and are able to provide real-time measurements. Moreover, they are easy to miniaturize and, in most cases, require minimum or no sample preparation [10]. In this context, significant efforts have been recently made to develop wearable and non-invasive biosensors for in vivo monitoring of complex samples [11,12,13]. The combination of new communication technologies, such as smartphones and tablets, with these bioanalytical devices allow the development and acquisition of easy-to-use devices and easy-to-read measurements [14,15].

The main challenge for commercial bioanalytical devices commonly lies in the material used as an interface. This material must be biocompatible or able to generate a bio-friendly environment, have a high surface area, be easy to manufacture, and, for electrochemical biosensors, have intrinsic conductivity [16]. In addition, when the target analyte is a large biomolecule, such as a nucleic acid or protein, additional challenges exist related to surface fouling and non-specific adsorption of molecules at its interface [17]. Fouling is defined as spontaneous accumulation of solids on the biosensor surface [18]. These solids result in blocking, which obstructs the flow of analyte and products from the matrix of the measurement solution to the bioreceptor, and vice versa. Therefore, the biosensor sensitivity decreases, possibly causing interference, reducing the quantification reliability, and usually, negatively affecting the operational lifetime. To minimize these problems, different strategies can be applied, which are classified into passive and active approaches [19]. Active approaches are based on removing already-adsorbed fouling elements, and can be achieved using temperature-responsive materials [20], acoustic waves [21], or mechanical actuation [22]. By comparison, passive approaches are related to the prevention of incoming fouling. Passive techniques usually imply the use of polymers or hydrogels as protective barriers [23,24,25,26,27]. For example, polyhydroxyethyl methacrylate (pHEMA) is an excellent candidate for generating protective barriers against fouling because it is a hydrophilic polymer that exhibits resistance to nonspecific adhesion of proteins [28,29,30]. 

It is worth mentioning that to effectively manage quality control and perform quality assurance tests, it is critical to monitor specific targets with accuracy, precision, and robustness [31,32]. Unfortunately, the reliability of biosensors is usually limited. Commercial biosensors provide accuracy values around ±20% and require recalibration several times per day using standard solutions [33]. In addition, quantification by direct interpolation to a calibration curve can lead to matrix effect problems. For this reason, analytical methods that do not require a calibration curve for operation are an attractive alternative for this type of sensor. In some cases, the standard additions method is a viable methodology to mitigate matrix effects and improve accuracy in the quantification process [34,35,36].

Furthermore, classical analytical techniques, such as HPLC or spectrophotometry, are usually time consuming, have a high cost, require expert staff, and involve complex sample preparation methods. For this reason, the development of rapid, low-cost, and easy-to-perform methods remains an active research area. Electrochemical biosensors are one of the most attractive options in this field due to their promising characteristics that fulfil these market demands. 

In the present work, the analytical parameters of an electrochemical biosensor were evaluated following an electrochemical method for glucose quantification. Measurements were performed using an amperometric configuration based on covalent immobilization of glucose oxidase (GOx) onto an electrochemical platform. This platform was obtained by grafting PFM (pentafluorophenyl methacrylate) onto tailored plasma polymerized HEMA-co-EGDA (ppHEMA-co-EGDA) hydrogel, which coats a titanium dioxide nanotubes array (TiO_2_NTAs). The biosensor architecture offers several benefits for its analytical performance, such as the high surface area of TiO_2_NTAs, which increases sensitivity; biocompatibility; covalent immobilization of GOx via amide bond formation, which minimizes leakage of enzymes from the biosensor structure; and the protective film of chitosan, which increases the long-term stability.

## 2. Materials and Methods

### 2.1. Materials

Titanium (99.7%), GOx type VII from *Aspergillus niger*, low molecular weight chitosan, and hydroxyethyl methacrylate (HEMA) were all supplied by Sigma Aldrich. Pentafluorophenyl methacrylate (PFM) was supplied by Apollo Scientific, and Argon 5.0 was supplied by Carburos Metaálicos. 

### 2.2. Biosensor Preparation

**Figure 1:**
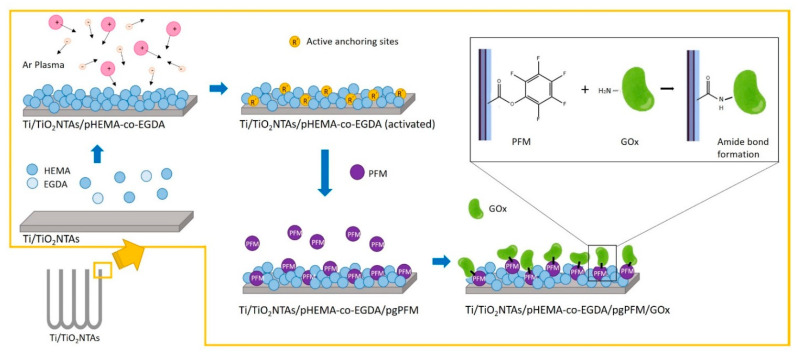
Scheme showing the covalent immobilization process. First, a plasma modification technique was used to obtain ppHEMA-co-EGDA/pgPFM film. Then, covalent immobilization of GOx was undertaken via amide bond formation.

First, Ti/TiO_2_NTAs was obtained by anodic oxidation (applied voltage 30 V for 17 h). The anodization process is explained in more detail in reference [37]. Then, the PFM plasma grafted surface was performed using an initial film deposition of HEMA-co-EGDA. This process is described in more detail in a previous work [38]. Briefly, plasma modification techniques were applied to the Ti/TiO_2_NTAs electrode to obtain a polymerized film of HEMA-co-EGDA and a grafted deposition of PFM. All the plasma modification were carried out in a stainless-steel vertical plasma reactor (base pressure about 9 × 10^−4^ mbar). To obtain the ppHEMA-co-EGDA film, the reactor was fed with approximately twice as much monomer (HEMA, 3.6 × 10^−2^ mbar) as cross-linker (EGDA, 1.2 × 10^−2^ mbar), and plasma polymerization was performed (20 W, duty cycle 2/22) for 10 min. Then, the ppHEMA-co-EGDA film was activated by Ar plasma and PFM was grafted at 7.7 × 10^−2^ mbar for 15 min. Thus, Ti/TiO_2_NTAs/ppHEMA-co-EGDA/pgPFM electrodes were obtained. Finally, GOx was immobilized by placing 20 µL of a GOx solution (15 mg/500 µL) on the modified PFM/grafted film. Figure 1 shows a schematic representation of the covalent immobilization process applied to attach the glucose oxidase molecules to the TiO_2_NTAs.

When the biosensors were not in use, they were immersed in 0.1 M PBS (pH 7.0) at 4 °C.

### 2.3. Electrochemical Measurements

**Figure 2:**
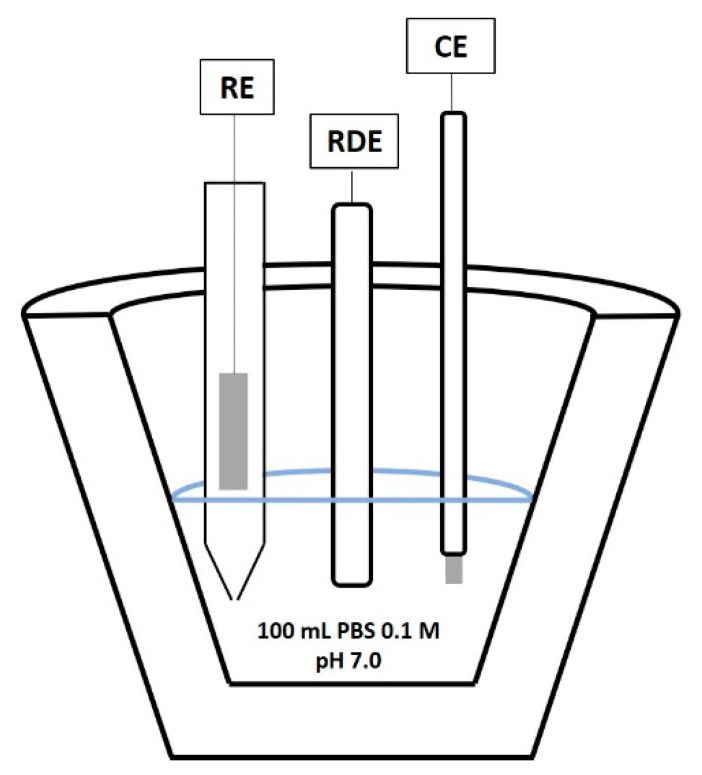
Schematic representation of the experimental setup.

Electrochemical measurements were performed using a Autolab 302N potentiostat/galvanostat and a standard three-electrode configuration (RE Ag/AgCl/3 M KCl and CE Pt foil). The working electrode was mounted in an RDE system (EG&G PARC model 616). The voltage applied to the biosensor was set to −0.4 V vs. Ag/AgCl/3 M KCl reference electrode, and amperometric measurements were performed at 2000 rpm. A schematic representation of the experimental setup is shown in Figure 2.

To perform the measurements, 100 mL of PBS 0.1 M (pH 7.0) was added to the measuring cell and the flowing current was measured continuously.

### 2.4. Glucose Determination in Food Samples

Glucose was determined using the standard addition method, in a set of different samples, using the developed biosensor. For this purpose, the current was continuously measured by applying a constant potential of −0.4 V vs. ref. Under these conditions, a blank signal corresponding to 100 mL of 0.1 M PBS (pH 7.0) was registered. After 2 min, the required amount of sample was placed without farther preparation into the measuring vessel. Finally, two consecutive glucose additions of a standard solution were made.

HPLC measurements were performed using the procedure described by the AOAC Official Method [39]. Samples for HPLC measurements were prepared as described in our previous work [37,38]. 

## 3. Results and Discussion

Glucose biosensors were prepared by covalent immobilization of GOx using PFM as an enzyme anchor via amide bond formation [40,41,42,43]. It was demonstrated in previous work that when PFM plasma was grafted onto an organic film of plasma polymerized HEMA-co-EGDA, the immobilized GOx molecules presented conformational active structures [38]. In previous work, the chemical reaction between PFM groups (plasma polymerized and grafted) was extensively studied, demonstrating the ability of PFM to react with amines, using IRRAS spectroscopy [44]. Thus, plasma grafting allowed high enzymatic activity of GOx on the electrode surface to be maintained.

Glucose measurements were indirectly made by reducing hydrogen peroxide to water at the electrochemical interface (Ti/TiO_2_NTAs). H_2_O_2_ was generated by the enzymatic conversion of glucose to gluconic acid in the presence of oxygen (see Reactions (1) and (2)):


Glucose + O_2_ → H_2_O_2_ + Gluconic Acid (1)

H_2_O_2_ + 2 H^+^ + 2e^−^ → 2H_2_ (2)

In previous work, using a similar biosensor architecture, the applied working potential was determined to be −0.4 V vs. the reference electrode (Ag/AgCl/KCl 3M) [37]. In this manner, classical reductive interferents, such as ascorbic acid or citric acid, can be avoided. In the present work, it was assumed that the working potential was not affected by the PFM plasma grafting. This modification technique generates isolated brushes of PFM in the electrochemical interface (TiO_2_NTAs/Ti), which should not alter the electrochemical capacity of the biosensor following the reduction of H_2_O_2_ to H_2_O [38]. 

The linear range, limit of detection (LOD), and limit of quantification (LOQ) of the Ti/TiO_2_NTAs/ppHEMA-co-EGDA/pgPFM/GOx/Chitosan biosensor were reported in a previous work [38]. The obtained values were: linear range from 0.25 to 1.49 mM, sensitivity 9.76 µA·mM^−1^, LOD 0.10 mM, and LOQ 0.20 mM. In addition, in the present work, the sensitivity of three different biosensors was measured, in addition to LOD and LOQ in the same linear range (from 0.25 to 1.49 mM glucose). The data are shown in Table 1.

Table 1 shows that differences between replicates were obtained (sensitivity, LOD, and LOQ). The average sensitivity was 8.53 ± 2.39 µA·mM^−1^. The variation of the sensitivity values is attributed to the complex construction process of the biosensor. However, it should be noted that this variation does not affect the sample quantification because it is performed using the standard addition method.

In the present work, we undertake in-depth evaluation of the sensor’s analytical performance. 

### 3.1. Evaluation of the Biosensor Enzymatic Activity

**Figure 3:**
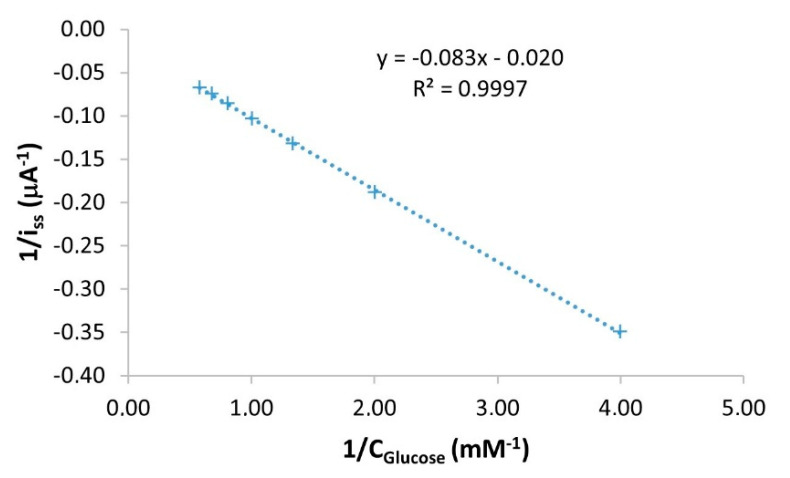
Representation of the Lineweaver-Burk equation using the data obtained from the calibration curve acquired using a Ti/TiO_2_NTAs/ppHEMA-co-EGDA/pgPFM/GOx/Chitosan biosensor.

The enzymatic activity of immobilized enzymes is an important parameter for evaluating the development processes of new biosensors. The greater the enzymatic activity, the better the biosensor performance. An indicator of the enzymatic activity exhibited by enzyme molecules is the Michaelis–Menten constant (K_M_), which is defined as the concentration of substrate necessary for the rate of the enzymatic reaction to reach a value equal to half the maximum rate. The lower the K_M_ value, the greater the ability of the substrate to bind to the active center of the enzyme and, therefore, the greater the catalytic activity of the enzyme [45]. By performing amperometric determinations, it is possible to relate the electrochemical information of the biosensors to the enzyme activity; to do this, the apparent Michaelis−Menten constant (K_M_^app^) is estimated using the Lineweaver−Burk equation (Equation (3)) [46].

(3) $$\frac{1}{i_{\mathrm{ss}}}=\frac{1}{i_{\max}}+\frac{k_{M}^{\mathrm{app}}}{i_{\max}}\cdot \frac{1}{C_{\mathrm{glucose}}}$$

where i_ss_ (µA) is the steady-state response current after the addition of the substrate, i_max_ (µA) is the maximum current under saturated substrate conditions, and C_glucose_ (mM) is the bulk glucose concentration. Therefore, by plotting the inverse of the substrate concentration vs. the inverse of the corrected intensity, a straight line with slope K_M_^app^/i_max_ and with the intercept equal to 1/i_max_ is obtained. A calibration curve obtained with the Ti/TiO_2_NTAs/ppHEMA-co-EGDA/pgPFM/GOx/Chitosan biosensor is used to obtain this information, and is presented in Figure 3.

As shown in Figure 3, the obtained linear regression equation using the data from the calibration curve was 1/i_ss_ (µA^−1^) = −0.083·1/C_Glucose_ (mM^−1^) − 0.020. Under the conditions of this study, K_M_^app^ of Ti/TiO_2_NTAs/ppHEMA-co-EGDA/pgPFM/GOx/Chitosan biosensors was estimated to be 4.20 mM. This determination was repeated using three different Ti/TiO_2_NTAs/ppHEMA-co-EGDA/pgPFM/GOx/Chitosan biosensors to measure its deviation. The resulting average K_M_^app^ value was 3.44 ± 0.67 mM with a relative standard deviation (RSD%) equal to 20%. This variation can be associated with the construction process in which several critical steps, such as the TiO_2_NTAs synthesis or plasma modification steps, can induce slight differences in the GOx immobilization and distribution. The obtained K_M_^app^ value was compared with others reported in the literature for similar glucose biosensors (see Table 2).

Table 2 shows that the Ti/TiO_2_NTAs/ppHEMA-co-EGDA/pgPFM/GOx/Chitosan biosensor yielded a low K_M_^app^ value (3.44 ± 0.67 mM) compared with others reported in the literature. This K_M_^app^ value implies a strong ability of substrate binding and high enzymatic activity of the immobilized enzyme molecules. Hence, the biosensors developed in the present work offer a greater affinity towards glucose than those listed in Table 2. This can be related to the enzymatic immobilization process that generates a biocompatible microenvironment for GOx molecules. This specific microenvironment offers adequate pH conditions and low hydrophobicity, and allows diffusion of reagents and products to/from the active center of the enzyme, blocking the passage of interferents. For these reasons, biosensors developed in the present work can maintain excellent catalyst activity once GOx molecules are immobilized.

### 3.2. Evaluation of Accuracy

Accuracy is the ability of the analytical method to provide values close to a given reference value. One of the simplest approaches to determine accuracy is to calculate the deviation between the obtained results using the tested method and a reference method. 

In the present work, glucose concentration of a commercial orange soft drink (test sample) was determined using high-performance liquid chromatography (HPLC) as the reference method and Ti/TiO_2_NTAs/ppHEMA-co-EGDA/pgPFM/GOx/Chitosan biosensor as the tested method. 

**Figure 4:**
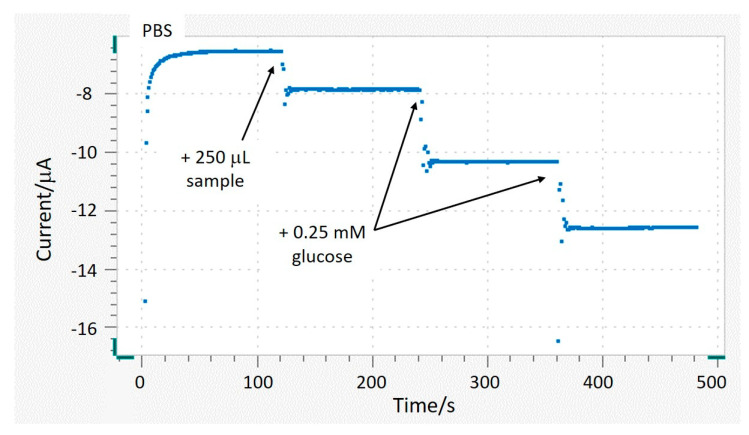
Current-time plot of the Ti/TiO_2_NTAs/ppHEMA-co-EGDA/pgPFM/GOx/Chitosan biosensor with an applied potential of -0.4 V when 250 mL of sample and two additions of 0.25 mM glucose injections were made.

The standard addition method was used to quantify glucose using the developed biosensor. The amperometric response of the Ti/TiO_2_NTAs/ppHEMA-co-EGDA/pgPFM/GOx/Chitosan biosensor was continuously monitored by applying −0.4 V vs. ref. at 2000 rpm. The blank signal was registered for 2 min (100 mL of PBS). Then, 250 µL of sample was added to the measuring vessel. Finally, two additions of 0.25 mM glucose standard were made. Figure 4 shows the measured current over time after each addition.

As shown in Figure 4, the registered current after the sample addition increased by approximately 1.3 µA, whereas after each standard injection (0.25 mM glucose) the current increment was about 2.2 µA. These current increments resulted from the reduction of H_2_O_2_ to H_2_O, which occurred due to the enzymatic reaction (see Reactions (1) and (2)). The additions curve was derived by plotting the corrected current vs. the added glucose concentration. The glucose content in the sample was quantified by extrapolation in this addition curve. 

Glucose concentration in the test sample was initially determined by HPLC. Measurements were performed using two replicates. Glucose determination using the biosensor was performed using three replicate measurements. The obtained results are shown in Table 3.

Table 3 shows that the obtained glucose concentration in the test sample determined by HPLC was 0.251 ± 0.001 M. The glucose content when the biosensor was used was 0.263 ± 0.005 M. The deviation between the two values was 4.8%. The Food and Drug Administration (FDA) established that a new bioanalytical method is accurate when the deviation between the obtained value and the reference value is below 15% [50]. In this work, the deviation was 4.8%, lower than the limit specified by the FDA. The obtained results demonstrate that Ti/TiO_2_NTAs/ppHEMA-co-EGDA/pgPFM/GOx/Chitosan biosensor was able to determine glucose concentration with sufficient accuracy.

### 3.3. Evaluation of Precision

Precision indicates the ability of the analytical method to deliver the same results when performing different measurements. To evaluate the precision of the method, repeatability and reproducibility were evaluated. The Ti/TiO_2_NTAs/ppHEMA-co-EGDA/pgPFM/GOx/Chitosan biosensor was used to determine the glucose content in a soft drink (test sample). All determinations were carried out using the standard addition method. 

The repeatability of the analytical method was evaluated on the same laboratory session using the same biosensor. Three replicate measurements of the glucose content in the test sample were performed and results are shown in Table 4.

As shown in Table 4, the biosensor yielded an average glucose concentration equal to 0.263 ± 0.005 M with a relative standard deviation (RSD%) lower than the reference value established by the AOAC (1.9%) [51]. 

In addition, the method reproducibility was evaluated by measuring the glucose content of the test sample during three different laboratory sessions using the same biosensor. The obtained results can be seen in Table 5.

As shown in Table 5, the biosensor offered an average glucose concentration equal to 0.262 ± 0.003 M. In this case, the relative standard deviation (RSD%) of these measurements was lower than the reference value established by AOAC (4.0%) [52].

For the development of bioanalytical methods, the Food and Drug Administration stablished that the precision determined should not exceed 15% of the RSD [53]. The results obtained not only meet this criterion, but also the more restrictive criteria stablished by the AOAC [51,52]. Therefore, it is possible to affirm that Ti/TiO_2_NTAs/ppHEMA-co-EGDA/pgPFM/GOx/Chitosan biosensors can be used to determine the glucose concentration with sufficient precision.

### 3.4. Evaluation of Robustness

Robustness is a measure of the susceptibility of an analytical method to changes that might occur during routine analysis, and provides an indication of the method’s reliability during normal use. It also shows the ability to reproduce the analytical method under different circumstances without the occurrence of unexpected differences [54]. 

For the validation of bioanalytical methods, it is not necessary to determine the robustness; however, this can be helpful to anticipate problems that may occur during the measurements [53]. In the present work, robustness was tested to anticipate potential problems related to the construction process of the biosensor that can affect the final glucose quantification. In this context, three different Ti/TiO_2_NTAs/ppHEMA-co-EGDA/pgPFM/GOx/Chitosan biosensors were constructed and then used to quantify the glucose content in the test sample on three different days. The obtained results are shown in Table 6.

Table 6 shows that the average glucose concentration obtained in the robustness test was 0.266 ± 0.006 M with an RSD of 2.4%. Because the RSD does not exceed 15% [53], it is possible to affirm that the quantification method is robust. Consequently, the manufacturing steps of Ti/TiO_2_NTAs/ppHEMA-co-EGDA/pgPFM/GOx/Chitosan biosensors do not have an influence on the glucose quantification process. 

### 3.5. Evaluation of Long-Term Stability

**Figure 5:**
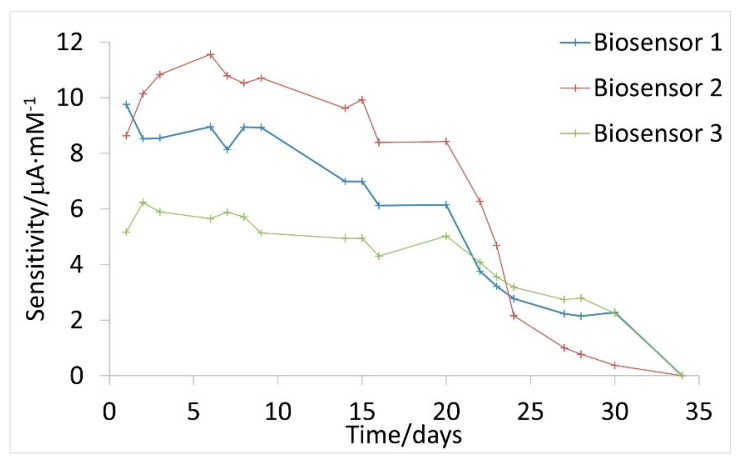
Evaluation of long-term stability for three Ti/TiO_2_NTAs/ppHEMA-co-EGDA/pgPFM/GOx/Chitosan biosensors.

Long-term stability is a pivotal requirement of a biosensor. This parameter defines the period during which the biosensor can be used with sufficient reliability. The long-term stability of the biosensors was evaluated by measuring its performance every few days; the slopes of calibration curves (related to sensitivity) obtained on several days were compared. Figure 5 shows the evolution over time of the sensitivity for three Ti/TiO_2_NTAs/ppHEMA-co-EGDA/pgPFM/GOx/Chitosan biosensors.

Figure 4 shows that sensitivity decreases over time. During the first 20 days following the construction of the biosensors, sensitivity showed slightly deviations for each biosensor. After 20 days, the developed biosensors conserved 89% of their initial sensitivity. However, after 20 days, the decrease in sensitivity became more pronounced. Finally, after 30 days the decrease in sensitivity was greater than 80% compared with the initial value in all cases.

A decrease in sensitivity is caused by enzyme deactivation, which can occur for several reasons. First, it should be noted that hydrogen peroxide generated during the enzymatic reaction can damage the enzyme molecules. H_2_O_2_ is a highly oxidizing compound that can react with some of the amino acids present in the structure of glucose oxidase [55,56], causing modifications to its structure and affecting its catalytic activity. Second, pH can also alter the structural conformation of the immobilized enzymes. Even maintaining pH 7.0 using a 0.1 M PBS solution, local significant pH variations may occur inside the nanotubes because of the reduction of H_2_O_2_ to H_2_O. These local pH alterations can affect the inter- and intramolecular bonds that generate the active structure of the enzyme, causing its deactivation [57]. Finally, the degradation of chitosan is another factor to be considered. Chitosan acts as a protective film. As time passes, this film is degraded. Consequently, enzyme molecules are more exposed to the measurement medium. Thus, as chitosan degrades, GOx can more easily lose its biologically active conformation. It is reported that chitosan films are stable for a period of four weeks stored at 4 °C [58], which is in concordance with the long-term stability of the developed biosensors. It must be noted that, in the present work, biosensors were immersed in 0.1 M PBS (pH 7.0) at 4 °C when were not in use.

It should also be noted that the three evaluated Ti/TiO_2_NTAs/ppHEMA-co-EGDA/pgPFM/GOx/Chitosan biosensors showed different sensitivities. This difference is associated with the construction process; the anodization process to obtain the electrochemical interface and surface plasma modification involves several steps that can influence the final active area of the electrochemical interface. Equally, it is possible that the population of enzymes in their biologically active conformation differs slightly between biosensors. Thus, a certain variation between sensitivity values can be obtained. It should be noted that, despite the differences observed between biosensors, the glucose quantification is not compromised because the quantification is carried out by the standard addition method, and not by direct interpolation of a calibration curve. This fact was previously demonstrated in the robustness study (RDS 2.4%).

### 3.6. Food Sample Quantification

It was demonstrated that the Ti/TiO_2_NTAs/ppHEMA-co-EGDA/pgPFM/GOx/Chitosan biosensors developed in the present work met the quality standards required to quantify glucose with reliability. All analytical parameters tested were in good agreement with the highly demanding AOAC and FDA quality standards. To demonstrate the versality of the proposed bioanalytical method, the developed biosensors were used to quantify glucose in six different samples: two soft drinks, one soya sauce, one tomato sauce, one yoghurt, and one sweetened nuts beverage (Spanish horchata). These complex sample matrices were selected because they show classical quantification problems. For example, lactose and galactose present in dairy products are common interferents in glucose quantification by HPLC. Moreover, high protein and fat content can cause unspecific adhesion on the electrode surface that can lead to quantification errors. Soy and tomato sauces present high fat and protein concentrations. Other classical interferents for amperometric glucose quantification are ascorbic and citric acids, which are both usually present in soft drinks [59]. 

Samples were also analyzed using HPLC and the obtained glucose concentrations were considered to be reference values. Samples were analyzed using two replicate measurements by both techniques, HPLC and amperometric biosensors. Glucose concentrations in each sample and their standard deviations (s), in addition to the deviation between the two methods, are shown in Table 7. 

The results obtained by amperometric measurements using the biosensors were in good agreement with HPLC reference values (see Table 7) because the deviation from the reference value was lower than 15% in all cases (the standard established for bioanalytical methods [53]). Therefore, the constructed biosensors showed excellent analytical properties and the proposed quantification method allowed glucose to be determined in complex samples with adequate sensitivity, accuracy, precision, and robustness. Moreover, the described biosensor system offers the advantages of short analysis time, and the capability to be repeated due to long-term stability.

Furthermore, it should be noted that the intrinsic structure of the Ti/TiO_2_NTAs/ppHEMA-co-EGDA/pgPFM/GOx/Chitosan biosensors developed in this work can be of special interest for clinical diagnostic and in vivo/real time measurements:

- Electrochemical interfaces based on titanium and titanium (IV) oxide promote the biocompatibility of the system; titanium is a pharmacologically inert metal that does not cause allergic reactions in the immune system and the human body does not reject it. Moreover, TiO_2_NTAs offers a high surface area and the ability to promote charge transfer processes. Thus, Ti/TiO_2_NTAs is an excellent electrochemical interface for implantable biosensors.
- Chitosan acts as a protective barrier for the immobilized enzyme and provides conformational stability to the measurement device. Chitosan is a biocompatible hydrogel obtained from natural sources that prevents enzyme denaturalization because of its high affinity for proteins [60]. Moreover, chitosan preserves its structure under adverse conditions due to its high mechanical stability. For this reason, using chitosan, the long-term stability of the biosensors is improved. The use of chitosan also improves the biosensor’s sensitivity because this polymeric matrix blocks possible interfering macromolecules.
- Poly-HEMA protects the electrochemical interface from adhesion of molecules that can interfere with the analytical measurement and/or cause damage to the human body, generating a foreign body response [61]. Therefore, the risk of rejection of implantable devices is reduced by using this polymer in the biosensor’ architecture. Furthermore, by using plasma techniques for HEMA polymerization, the obtained films follow the shape of the electrochemical interface [62]. Thus, ppHEMA-co-EGDA films follow the architecture of the TiO_2_NTAs, and both high specific surface area and high sensitivity are maintained.
- Covalent immobilization of enzyme molecules provides several benefits. First, it decreases the probability of enzyme leakage from the biosensor architecture. Second, covalent configuration ensures that enzymes are located close enough to the transducer to avoid the loss of the electrons produced from the enzymatic reaction. In addition, the pgPFM surface shows high covalency, which is the ability of the surface to retain the attached molecules after vigorous washing [63]. Hence, the developed biosensors are likely to retain their sensitivity under milder conditions, such as those found in the human bloodstream. Finally, the PFM plasma-grafting modification minimizes enzyme deactivation during the immobilization process [38].

It should also be noted that in our previous work [37] we used polymer entrapment as an enzyme immobilization method in the sensor architecture. In contrast, in the present work the enzyme immobilization process was changed to covalent binding (pgPFM and HEMA-co-EGDA). An advantage of this method is that, because of the stable nature of the bonds formed between the enzyme and matrix, the enzyme is not released into the solution upon use. In addition, when covalent binding is used, the enzyme is strongly bound to the matrix and is also stabilized [64]. Furthermore, GOx molecules adopt biological active conformations (native and molten globule structures) when immobilized in the pgPFM and HEMA-co-EGDA layer. This was demonstrated using QCM-D [38]. For this reason, it was possible to increase the enzymatic capacity of the biosensor and, as a result, a higher sensitivity than that of our previous work [37] (5.46 µA·mM^−1^) was obtained (9.76 µA·mM^−1^).

Therefore, Ti/TiO_2_NTAs/ppHEMA-co-EGDA/pgPFM/GOx/Chitosan biosensors showed outstanding analytical properties due to the synergy of the electrochemical interface, the immobilization technique, and the protection matrix. In addition, because of this synergy, the proposed biosensors have significant potential for the development of implantable devices for biomedical applications.

## 4. Conclusions

The analytical parameters of Ti/TiO_2_NTAs/ppHEMA-co-EGDA/pgPFM/GOx/Chitosan biosensors were evaluated using a commercial soft drink as a test sample. The immobilized enzyme molecules on the biosensor showed a strong ability of substrate binding and high enzymatic activity, as indicated by the K_M_^app^ value of 3.44 ± 0.67 mM. In addition, measurements undertaken with this biosensor showed high accuracy, with a deviation from HPLC results equal to 4.8%. This biosensor offered excellent analytical parameters: high repeatability (RSD = 1.7%), high reproducibility (RSD = 1.3%), and high robustness (RSD = 2.4%). Finally, the long-term stability was further examined and after 20 days, the Ti/TiO_2_NTAs/ppHEMA-co-EGDA/pgPFM/GOx/Chitosan biosensors retained 89% of their initial sensitivity.

Moreover, glucose quantification was performed in complex samples, in which sample preparation only required dilution. For all of the studied samples, the obtained results were in good agreement with the HPLC reference values. In addition, the proposed biosensor was able to overcome typical sources of interference in glucose quantification, such us galactose, ascorbic acid, fats, and proteins. Therefore, the proposed biosensor can be considered to be an inexpensive, fast, and simple alternative to classical analytical quantification methods.

The exceptional analytical properties of this biosensor can be attributed to its intrinsic architecture. The unique electrochemical platform is a versatile interface that is biocompatible and promotes charge transfer processes, leading to increase sensitivity. The immobilization process, which consists of the formation of an amide bond, minimizes the loss of enzyme molecules. In conjunction with the use of chitosan as a protective barrier, this improves long-term stability. Finally, the presence of polyHEMA, which prevents nonspecific adhesion of proteins, ensures the biosensor has significant versatility. Due to the synergy of these features, the proposed biosensor is a good candidate for future applications in diagnostic, food, and environmental analysis.

## Figures and Tables

**Table 1:** Values of sensitivity, LOD, and LOQ obtained for several Ti/TiO_2_NTAs/ppHEMA-co-EGDA/pgPFM/GOx/Chitosan biosensors. The linear range was equal for all of the evaluated sensors.

Biosensors	Sensitivity/µA·mM^−1^		LOD/mM	LOQ/mM
	Individual Values	Average		
[38]	9.76	8.53 ± 2.39	0.10	0.20
Present work	8.63		0.12	0.20
	5.15		0.14	0.21
	10.58		0.10	0.19

**Table 2:** Values of the apparent Michaelis–Menten constant of different glucose biosensors described in the literature and of the Ti/TiO_2_NTAs/ppHEMA-co-EGDA/pgPFM/GOx/Chitosan biosensor developed in the present work. The value shown in the present work is the average value and its deviation obtained from three different biosensors.

Biosensor	K_M_^app^/mM	Reference
GCE-CNT/GOx	5.95–14.50	[45]
GCE/CS/CNT/Au-PtNPs/GOx	5.20	[47]
CNT/Pt/GOx/Nafion	10.11	[48]
Ti/TiO_2_NT/AuNPs/GOx	7.2	[49]
Ti/TiO_2_NTAs/ppHEMA-co-EGDA/pgPFM/GOx/Chitosan	3.44 ± 0.67	Present work

**Table 3:** Glucose determination using different analytical techniques on the same orange soft drink sample.

Method	[Glucose] ± s/M	RSD/%	Deviation/%
HPLC	0.251 ± 0.001	0.3	4.8
Biosensor	0.263 ± 0.005	1.7	

**Table 4:** Study of the system repeatability. Measurements were performed on the same day using the same Ti/TiO_2_NTAs/ppHEMA-co-EGDA/pgPFM/GOx/Chitosan biosensor.

Replicate	[Glucose]/M	[Glucose] Average ± s/M	RSD/%
1	0.260	0.263 ± 0.005	1.7
2	0.268		
3	0.261		

**Table 5:** Study of the system reproducibility. Measurements were performed on three different days using the same Ti/TiO_2_NTAs/ppHEMA-co-EGDA/pgPFM/GOx/Chitosan biosensor.

Day	[Glucose]/M	[Glucose] Average ± s/M	RSD/%
1	0.266	0.262 ± 0.003	1.3
2	0.261		
3	0.260		

**Table 6:** Study of the system robustness. Measurements were performed on three different days using three different Ti/TiO_2_NTAs/ppHEMA-co-EGDA/pgPFM/GOx/Chitosan biosensors.

Day	Biosensor	[Glucose]/M	[Glucose] Average ± s/M	RSD/%
1	1	0.258	0.266 ± 0.006	2.4
2	2	0.270		
3	3	0.268		

**Table 7:** Glucose concentration values obtained using the Ti/TiO_2_NTAs/ppHEMA-co-EGDA/pgPFM/GOx/Chitosan biosensor and HPLC values. Standard deviation (s) and deviation of both methods are also shown.

Sample	[Glucose]_biosensor_ ± s/M	[Glucose]_HPLC_ ± s/M	Deviation %
Orange Soft Drink	0.263 ± 0.005	0.251 ± 0.001	4.8
Lemon Soft Drink	0.164 ± 0.001	0.150 ± 0.001	9.1
Soya Sauce	0.087 ± 0.002	0.096 ± 0.001	−9.7
Tomato Sauce	0.559 ± 0.009	0.515 ± 0.005	8.4
Yoghurt	0.188 ± 0.002	0.174 ± 0.001	8.1
Horchata	0.038 ± 0.002	0.035 ± 0.001	8.5

## Data Availability

Not applicable.
